# The neurobiological regulatory mechanism of brain edema

**DOI:** 10.3389/fncel.2026.1777039

**Published:** 2026-03-31

**Authors:** Meiqi Tian, Man Li, Chaoying Zhang, Qi Luo, Aiwen Wang, Dongyang Li

**Affiliations:** 1 School of Stomatology, Hainan Medical University, Haikou, China; 2 Department of Cardiology, Yan’an Hospital Affliated to Kunming Medical University, Kunming, China; 3 The First Clinical College, Hainan Medical University, Haikou, China; 4 Department of Neurological Intensive Care Unit, Daqing Longnan Hospital, Daqing, China; 5 Key Laboratory of Brain Science Research Transformation in Tropical Environment of Hainan Province and Key Laboratory of Tropical Translational Medicine of Ministry of Education, College of Basic Medical Sciences, Hainan Medical University, Haikou, China

**Keywords:** blood–brain barrier, brain edema, inflammatory, neurobiology, signaling pathways

## Abstract

Cerebral edema is a common pathological condition associated with a variety of neurological disorders, and its development involves numerous neurobiological mechanisms. This review explores the neurobiological regulatory mechanisms underlying cerebral edema, including the disruption of the blood–brain barrier, inflammatory responses, alterations in vascular permeability, and intracellular edema. We will investigate the formation mechanisms of cerebral edema under different pathological states and discuss potential therapeutic strategies, aiming to provide insights for clinical treatment. Current research highlights the complexity of the interactions between these mechanisms and the need for targeted interventions to mitigate the impact of cerebral edema on patient outcomes. This review aims to synthesize existing knowledge and encourage further exploration in this critical area of neuroscience, ultimately contributing to more effective management of cerebral edema.

## 1 Introduction

Cerebral edema, defined as the accumulation of excess fluid in the brain tissue, leads to an increase in brain volume and can result in elevated intracranial pressure (ICP) and neurological dysfunction. The pathophysiology of cerebral edema is multifaceted, involving various mechanisms such as cellular fluid transport, inflammatory responses, and neuroregulatory processes. Recent advancements in our understanding of these neurobiological regulatory mechanisms have significantly enhanced our comprehension of underlying causes and potential therapeutic targets of cerebral edema. This review aims to explore the neuroregulatory mechanisms involved in cerebral edema formation and discuss their implications for clinical management and treatment strategies.

The formation of cerebral edema can be categorized into different types, including cytotoxic, vasogenic, and osmotic edema, each with distinct underlying mechanisms. Cytotoxic edema primarily results from cellular injury, leading to the failure of ion pumps and subsequent cellular swelling. Vasogenic edema, on the other hand, occurs due to the disruption of the blood–brain barrier (BBB), allowing plasma proteins and fluid to leak into the extracellular space, thus increasing interstitial fluid volume (Chen S. et al., 2021). Inflammatory processes play a critical role in both cytotoxic and vasogenic edema, as the release of pro-inflammatory cytokines can exacerbate BBB dysfunction and promote fluid accumulation (Wang et al., 2025). Understanding these mechanisms is crucial for developing targeted therapies that can mitigate the effects of cerebral edema and improve patient outcomes.

Recent advances in the signaling pathways involved in cerebral edema formation have opened new avenues for therapeutic interventions. For instance, targeting specific molecules like matrix metalloproteinase-9 (MMP-9) has shown promise in reducing BBB disruption and edema in preclinical models of traumatic brain injury (TBI) (Sunny et al., 2024). Additionally, the use of thrombin inhibitors has been suggested as a strategy to alleviate edema following cortical injuries, underscoring the potential of pharmacological agents to influence the progression of edema development (Singh et al., 2025). Furthermore, the emergence of innovative drug delivery systems, such as nanocarriers, may improve the targeting of therapeutic agents to the inflamed brain, which could enhance the management of cerebral edema (Ollen-Bittle et al., 2024).

In conclusion, cerebral edema remains a significant clinical challenge, with complex neurobiological foundations that require a comprehensive understanding of its regulatory mechanisms. As research continues to demonstrate the intricate interactions between cellular, molecular, and inflammatory factors contributing to edema formation, there is potential for the development of novel therapeutic strategies aimed at improving patient care. This review will serve as a foundation for further exploration into the neuroregulatory mechanisms of cerebral edema and their impact on clinical practice.

## 2 Types of brain edema

### 2.1 Cytotoxic edema

Cytotoxic Edemais characterized by intracellular swelling of neurons, glia, and endothelial cells due to failure of the ATP-dependent sodium-potassium pump, typically following ischemic or metabolic insults (Manwar et al., 2023). This results in intracellular accumulation of sodium and water. A critical neurochemical event associated with this process is the release of ascorbate, which has been directly observed during glutamate-induced cytotoxic edema (Jin J. et al., 2020). The swelling of astrocytic endfeet is an early event, preceding measurable breakdown of the BBB (Manwar et al., 2023). The aquaporin (AQP) 4 water channel, predominantly located on astrocytic endfeet, plays a pivotal role in facilitating this water influx (Contreras-Zarate et al., 2023). Resolution of cytotoxic edema depends on the restoration of ionic homeostasis. Pharmacological strategies aim to block the initiating cascades; for instance, the anti-epileptic drug topiramate, which inhibits AQP4 function, can reduce radiation-induced astrocytic swelling and cytotoxic edema (Contreras-Zarate et al., 2023). Similarly, genetic knockout or pharmacological inhibition of the sulfonylurea receptor 1 (SUR1), which regulates the SUR1-TRPM4 channel involved in oncotic cell swelling, has been shown to ameliorate cytotoxic edema and improve outcomes in models of sepsis and traumatic brain injury (Moosavi et al., 2016).

### 2.2 Vasogenic edema

Vasogenic Edema arises from increased permeability of the BBB, allowing plasma proteins and fluid to extravasate into the extracellular space. This is often triggered by conditions that impair endothelial integrity, such as hypertension, inflammation, or certain tumors (Li Y. et al., 2023). The disruption is multifocal and initiates around capillaries and venules (Hoffmann et al., 2018). Key mediators include proteolytic enzymes like proteinase-3, which can degrade endothelial junctional proteins (Cummings et al., 2023). In conditions like posterior reversible encephalopathy syndrome, vasogenic edema is a hallmark, believed to stem from endothelial dysfunction and hyperperfusion (Li Y. et al., 2023). Resolution requires restoration of BBB integrity, which involves downregulation of inflammatory mediators and reparative processes in the endothelium. The extent of vasogenic edema can predict subsequent tissue injury, as its volume correlates with final infarct size after ischemia (Hoffmann et al., 2018). It is important to differentiate vasogenic edema from other types using advanced imaging; for example, in cerebral venous thrombosis, vasogenic edema and hemorrhage are more common consequences than true venous infarction (Alajmi et al., 2023).

### 2.3 Hydrostatic edema

Hydrostatic Edema occurs when increased pressure within the cerebral ventricles forces cerebrospinal fluid across the ventricular ependymal lining into the periventricular white matter. While this classic hydrostatic mechanism is well-established, the provided literature emphasizes that in pathological contexts like post-hemorrhagic ventricular dilatation, periventricular white matter injury involves complex microstructural changes including axonal injury, myelin disruption, and vasogenic edema, rather than pure interstitial fluid accumulation (Isaacs et al., 2022).

### 2.4 Ionic edema

Ionic Edema represents an intermediate stage in the edema cascade. It is defined by the accumulation of sodium and other ions in the extracellular space due to a compromised BBB ionic gradient, but before the extravasation of large plasma proteins (Biller et al., 2021). This subtype was recently demonstrated in healthy humans following prolonged normobaric hypoxia, where sodium MRI detected sodium ion accumulation in the extracellular space alongside an intact endothelium (Biller et al., 2021). This suggests a transitional state where the BBB may be functionally altered but not fully disrupted. The resolution of ionic edema likely involves the active export of ions from the interstitium, possibly via astrocytic and endothelial ionic pumps, to re-establish the trans-endothelial electrochemical gradient before progression to full vasogenic edema.

## 3 Causes of brain edema

**Figure 1:**
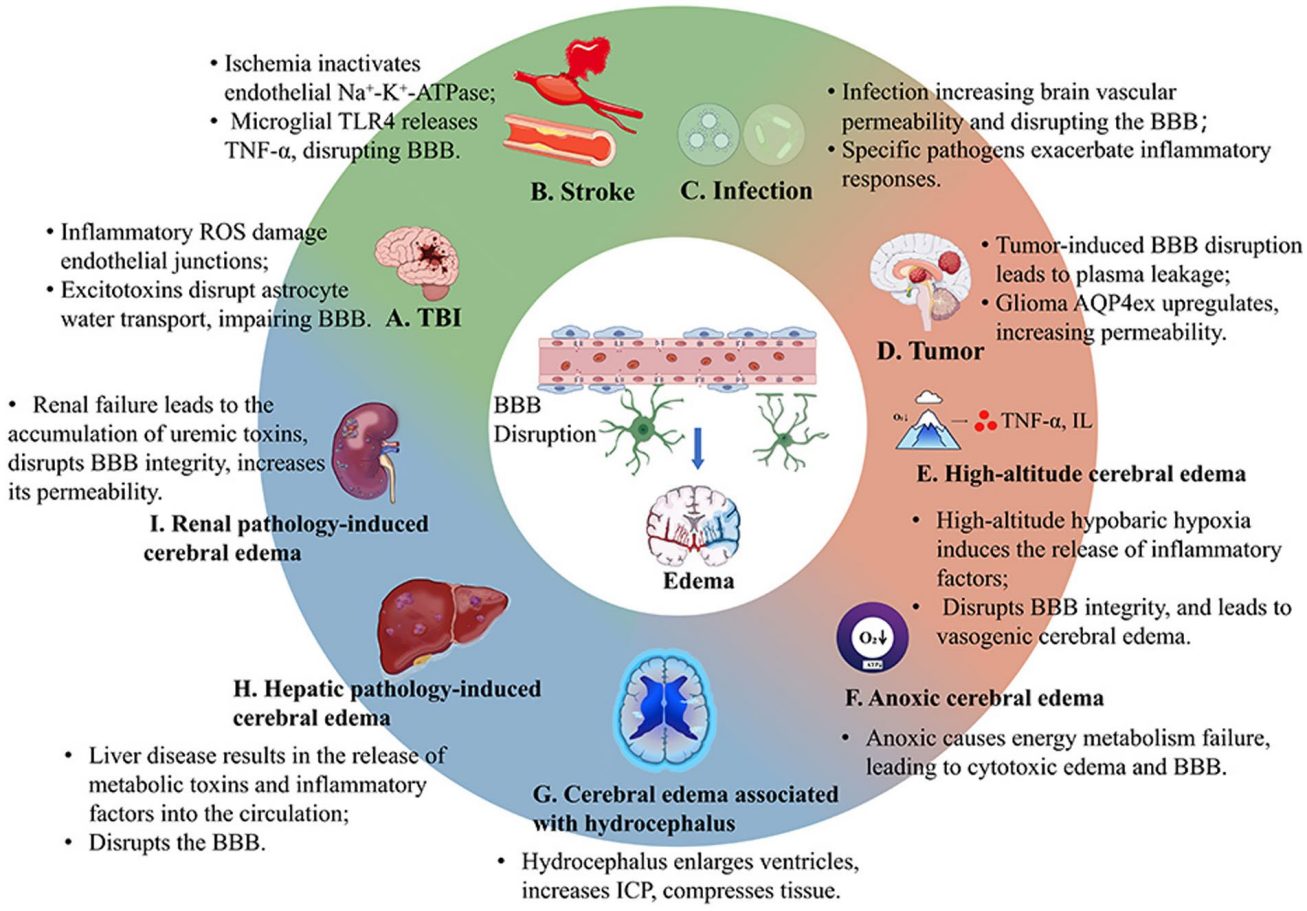
The mechanisms of cerebral edema caused by disruption of the blood–brain barrier under different pathological conditions. **(A–I)** TBI, stroke, infections, tumors, High-altitude cerebral edema, Anoxic cerebral edema, Cerebral edema associated with hydrocephalus, Hepatic pathology-induced cerebral edema, and Renal pathology-induced cerebral edema disrupting the BBB.

Brain edema, characterized by the accumulation of excess fluid in the brain, can arise from various pathological conditions. Understanding the causes is crucial for developing effective treatment strategies (Figure 1).

### 3.1 Traumatic brain injury

TBI is recognized as one of the leading causes of morbidity and mortality worldwide, with a significant impact on public health across all age groups. From infants to the elderly, individuals can sustain TBI due to a variety of incidents, including falls, motor vehicle accidents, sports injuries, and assaults. According to the World Health Organization, TBI affects millions of people globally each year, contributing not only to immediate fatalities but also to long-term disabilities affecting physical, cognitive, and psychosocial domains (Wiles, 2022). The effects of TBI extend beyond the impacted individuals to families and communities, imposing significant economic burdens on healthcare systems and societal structures.

TBI usually originates from a primary injury, directly related to an external blow to the brain. The common symptoms include loss of consciousness, confusion, headache, dizziness, sensory disturbances, and emotional changes. Neurological assessments often reveal a mix of cognitive impairment and motor dysfunction, with some individuals experiencing communication difficulties or altered coordination. The mechanisms behind TBI are complex and involve a cascade of pathological processes. Key mechanisms include inflammatory responses, disruption of the BBB, and excitatory amino acids. Following injury, inflammatory cells infiltrate the brain tissue, releasing cytokines and reactive oxygen species that exacerbate neuronal damage (Kaur and Sharma, 2018). Degradation of the BBB is a critical event that leads to increased permeability, allowing potentially harmful substances to enter the neural tissue and contributing to edema formation (Cash and Theus, 2020). Moreover, the release of excitatory amino acids exacerbates neuronal excitability and cell death, leading to sustained neurological dysfunction (Dorsett et al., 2017).

Cerebral edema, a significant complication arising from TBI, is characterized by an abnormal accumulation of fluid within the brain parenchyma. Perihemorrhagic edema is common in TBI patients, which can cause increased ICP, hydrocephalus, and even cerebral herniation, which may lead to permanent brain damage and death (Sulhan et al., 2020). The interplay between trauma-induced cellular and molecular alterations promotes edema formation and can severely complicate the clinical management of TBI. Research has indicated that disturbances in water homeostasis in the brain are notably governed by AQPs (Mamtilahun et al., 2019; Czyzewski et al., 2024). Notably, AQP4 has been identified as a pivotal mediator in post-traumatic gliosis and cerebral edema following TBI (Czyzewski et al., 2024). Enhanced AQP4 expression is associated with increased water influx and subsequent edema, while inhibition or dysregulation of AQP4 can help mitigate the extent of edema formation. Additionally, the integrity of the BBB is central to the pathological development of TBI-related edema. Injury to the BBB can lead to neuroinflammation, exacerbating the permeability of the barrier and facilitating the development of edema. Recent studies have elucidated the role of various signaling pathways and inflammatory mediators in the disruption of the BBB following TBI (Lopez et al., 2022), highlighting potential therapeutic targets for intervention. This understanding reinforces the need for ongoing research focusing on the relationship between AQP expression, BBB integrity, and cerebral edema to facilitate the development of innovative strategies aimed at reducing morbidity associated with TBI.

In summary, TBI represents a complicated injury mechanism leading to diverse clinical presentations and multiple pathophysiological processes, including cerebral edema. Understanding the intricate interplay between inflammatory responses, BBB disruption, and the role of AQPs is crucial for developing future therapeutic approaches to mitigate the effects of TBI and improve outcomes for affected individuals. Future studies are warranted to explore targeted interventions that address these pathways to alleviate edema and enhance recovery in TBI patients.

### 3.2 Stroke

Stroke, a leading cause of death and long-term disability worldwide, can be classified into two major types: ischemic and hemorrhagic strokes. Ischemic stroke, the most common type, occurs when there is a blockage in the blood vessels supplying oxygen and nutrients to the brain. Hemorrhagic stroke, on the other hand, involves bleeding within the brain tissue, either from ruptured arteries or veins. Stroke can lead to severe consequences such as brain edema.

**Figure 2:**
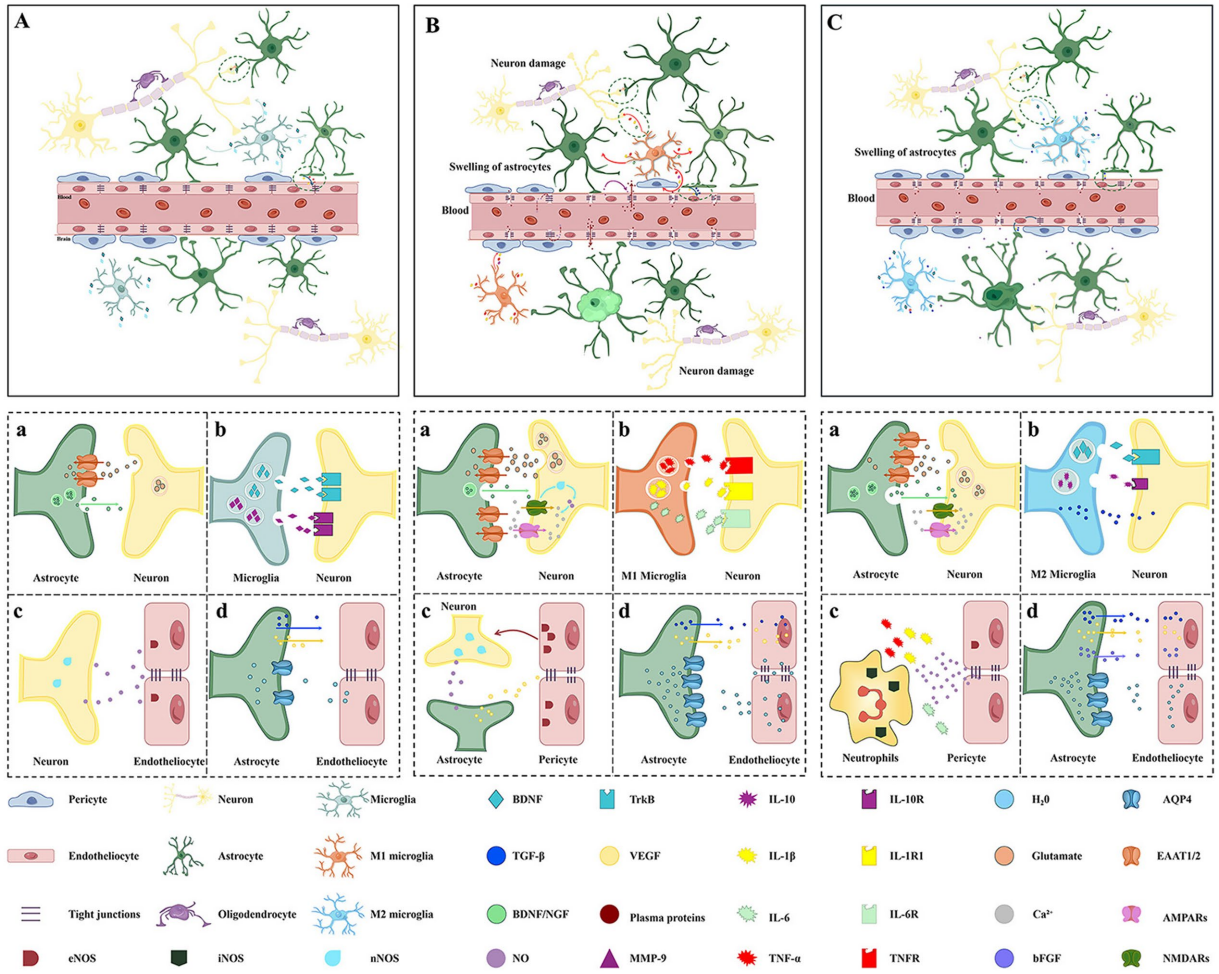
Neurobiological regulatory mechanisms after cerebral edema. **(A)** Structure of the normal neurovascular unit. The brain capillary is structurally composed of cerebral capillary endothelial cells bound together by tight junctions. Together with neurons (yellow in the image), astrocytes (dark green in the image), (purple in the image), and microglial cells (pale green in the image), they collectively ensure the integrity of the blood–brain barrier. **(B)** In the early stages of cerebral edema, neuronal damage activates microglia toward the M1 phenotype and induces astrocyte swelling, impairing glutamate uptake. Microglia generate excessive reactive oxygen species, exacerbating oxidative stress. Extracellular glutamate accumulation overstimulates neuronal NMDARs and AMPARs, causing Ca^2+^ overload and excitotoxicity. Subsequently, astrocytic endfeet retract from endothelial cells (**a,b**). nNOS-derived NO promotes VEGF synthesis in astrocytes. eNOS-produced NO supports neuronal function **(c)**. Endothelial MMPs degrade extracellular matrix and tight junctions. AQP4 expression is upregulated and undergoes relocalization, facilitating water influx into brain tissue and exacerbating cerebral edema **(d)**. **(C)** In the late stage of cerebral edema, astrocytes secrete BDNF, which activates neuronal TrkB receptors, promoting neuronal survival, axonal regeneration, and synaptic remodeling **(a)**. Microglia transform into the M2 phenotype. M2 microglia secrete anti-inflammatory cytokines such as TGF-β and IL-10, as well as neurotrophic factors like BDNF, participating in tissue repair and neural regeneration **(b)**. Neutrophils produce iNOS-derived NO, disrupting gap junctions, and release inflammatory mediators **(c)**. Astrocytes secrete VEGF and bFGF, stimulating endothelial proliferation and migration, while TGF-β helps stabilize tight junctions in nascent vessels, contributing to BBB recovery. AQP4 expression is downregulated, reducing water influx into brain tissue, alleviating edema and restoring brain water balance **(d)**. BBB, blood–brain barrier; TNF-α, tumor necrosis factor-α; NMDARs, N-methyl-D-aspartate receptors; MMPs, matrix metalloproteinases; AMPARs, α-amino-3-hydroxy-5-methyl-4-isoxazolepropionic acid receptors; AQP4, aquaporin 4; bFGF, basic fibroblast growth factor; EAAT1/2, excitatory amino acid transporters 1/2.

The brain edema plays a crucial role in the development and progression of stroke-related injuries. The neurovascular unit (NVU), which consists of endothelial cells, pericytes, astrocytes, and neurons, plays an essential role in maintaining the integrity of the BBB and regulating the movement of ions, water, and nutrients across it (Figure 2). In ischemic stroke, reduced cerebral blood flow (CBF) leads to changes in NVU transport proteins (e.g., N-methyl-D-aspartate receptors, NMDARs), resulting in increased permeability of the BBB and subsequent brain edema. This process also involves alterations in energy metabolism due to changes in glucose transport proteins (Sifat et al., 2019). As highlighted in recent studies, NMDARs are critical players in the pathophysiology of ischemic stroke. Overactivation of NMDARs during ischemia leads to excessive glutamate release, which triggers calcium overload and neuronal death (Figure 1B; Zong et al., 2022; Yu et al., 2023). Furthermore, the interaction between NMDARs and the NVU exacerbates BBB disruption, contributing to the severity of brain edema through disturb the intercellular tight junctions (Figure 2d; Ge et al., 2020; Yu et al., 2022). Understanding these mechanisms is pivotal for developing targeted therapies aimed at mitigating stroke-induced neurological damage.

Studies have highlighted Toll-like receptor 4 as a potential therapeutic target, with its inhibition showing notable benefits in stroked patients. Persistent inflammation mediated by Toll-like receptor 4 can lead to a range of associated diseases, including hydrocephalus and elevated ICP, vasospasm, microthrombosis, and BBB disruption (Shang et al., 2019). In addition to its effects on the BBB, stroke can also impact other aspects of brain function. For example, increased secretion of arginine-vasopressin (AVP) has been observed following stroke, which may contribute to the development of brain edema (Li D. et al., 2021; Cui et al., 2021). Moreover, stroke-induced brain edema has been linked with various diseases such as cerebral infarction and hemorrhage. Therefore, understanding the mechanisms underlying stroke-induced brain edema is crucial for developing effective treatment strategies.

In conclusion, understanding the complex interplay between the NVU, BBB, and cerebral edema in the context of stroke is essential for developing effective therapeutic strategies. Targeting specific components of this pathway, such as Toll-like receptor 4, offers promising avenues for reducing the debilitating effects of stroke and improving patient outcomes.

### 3.3 Infections

Infection refers to the local tissue and systemic inflammatory response caused by the invasion of pathogens such as bacteria, viruses, fungi, and parasites into the human body. Under normal circumstances, inflammation is a defensive response of the body that can promote tissue repair and functional normalization. Infections increase vascular permeability in the brain, predominantly resulting in vasogenic cerebral edema. The disruption of the BBB due to infection allows plasma constituents and leukocytes to enter the brain parenchyma, exacerbating inflammation and edema formation. Different types of infectious diseases causing brain edema (Table 1; Azevedo-Quintanilha et al., 2019; Krous et al., 2007; Yao et al., 2015; Bharath et al., 2023; Zhao et al., 2021; Song et al., 2016; Davenport et al., 1990; Kidney and Kim, 1998; van der Flier et al., 2005; Lin et al., 2023).

**Table 1:** Infectious diseases causing brain edema.

Infectious disease	Primary associated pathogen	Key pathogenic mechanisms and pathological features	References
Malaria	Plasmodium berghei ANKA (experimental model)	1. BBB disruption, astrocyte swelling, adhesion and accumulation of leukocytes in cerebral vessels, and a pro-inflammatory response are key factors. 2. Deficiency of integrin αDβ2 attenuates BBB disruption and cerebral edema.	Azevedo-Quintanilha et al. (2019)
Viral meningitis	Various viruses (e.g., enterovirus, HHV-6)	Even clinically mild viral (lymphocytic) meningitis can cause severe diffuse cerebral edema, brain herniation, and neurogenic pulmonary edema in young children.	Krous et al. (2007)
	Influenza virus (H1N1)	Infection increases BBB permeability and causes brain edema, associated with overexpression of inflammatory mediators such as endothelin-1, inducible nitric oxide synthase, and TNF-α.	Yao et al. (2015)
	SARS-CoV-2 (Omicron BA.2)	Infection in children can cause acute fulminant cerebral edema, often accompanied by shock and multi-organ failure; pathophysiology may be related to a significant systemic hyperinflammatory response.	Lin et al. (2023)
Ameba infections	*Naegleria fowleri*	Causes Primary Amebic Meningoencephalitis; neuroimaging may show a nonspecific pattern of diffuse cerebral edema.	Kidney and Kim (1998)
	Acanthamoeba spp.	Causes Granulomatous Amebic Encephalitis; neuroimaging often shows focal enhancing lesions.	
Bacterial meningitis	Pneumococcus	In an experimental model, VEGF levels in CSF were elevated and correlated with brain edema.	van der Flier et al. (2005)
Mycoplasma/ureaplasma infection	*Mycoplasma hominis*, Ureaplasma spp.	Causes disseminated infection in immunocompromised patients, leading to nonhepatic hyperammonemia syndrome, which can result in severe cerebral edema and refractory status epilepticus.	Bharath et al. (2023)
Other bacterial infections	Leptospira spp.	Patients with severe infection and multisystem involvement may develop cerebral edema during treatment for acute renal failure (e.g., hemodialysis or hemofiltration).	Davenport et al. (1990)
	*Mycobacterium tuberculosis*	Causes adrenal insufficiency, which can precipitate an adrenal crisis under stress, leading to diluted hyponatremia and severe cerebral edema.	Zhao et al. (2021)
Infection as a predisposing factor	Preexisting systemic inflammation/infection	Can significantly facilitate the development of cerebral edema upon subsequent exposure to short-term severe hypoxia (e.g., high altitude). The mechanism involves inflammatory signaling upregulating the expression and function of the brain water channel AQP4.	Song et al. (2016)

Infections are among the most common complications following ischemic stroke, further complicating patient outcomes. Post-stroke infections can significantly impact stroke prognosis by aggravating neuroinflammation. Peripheral inflammatory signals have been shown to communicate with the central nervous system (CNS), exacerbating neural inflammatory responses. The lipopolysaccharide-induced ischemic brain injury infection model led to an excessive inflammatory response, which consequently increased the infarct volume and edema degree 24 h after middle cerebral artery occlusion (Wang et al., 2019). Plasmodium infection can induce reversible brain swelling through disrupting BBB (Jin et al., 2022). This finding highlights the potential for parasitic infections to directly influence cerebral edema development. In C57BL/6 mice infected with toxoplasma gondii, vasogenic edema was observed. Treatment with a viral vector expressing vascular endothelial growth factor (VEGF) showed improvements in lymphatic drainage in infected mice (Kovacs et al., 2024). However, this treatment did not result in increased clearance of edematous fluid from the brain, suggesting complex interactions between infection, vascular responses, and edema resolution.

### 3.4 Tumors

Cerebral edema associated with brain tumors is a very common occurrence, including primary and metastatic tumors. Leakage of plasma across the vessel wall into the parenchyma through the disruption of the BBB, causes edema around the brain tumor, also known as brain tumor-related edema (Esquenazi et al., 2017; Solar et al., 2022). Since the brain is enclosed within the skull, an increase in ICP due to tumor growth or edema is initially countered by compensatory mechanisms such as cerebrospinal fluid displacement, reduced CBF, and alterations in brain parenchyma shape. However, as the tumor progresses, these compensatory mechanisms weaken, leading to a dramatic rise in ICP (Sorribes et al., 2019). The high ICP can paradoxically inhibit the production and circulation of CSF, potentially leading to the accumulation of toxic proteins, solutes, pro-inflammatory cytokines, and chemokines, exacerbating glioma progression (Ma et al., 2019). Furthermore, the expression of AQPs plays a pivotal role in gliomas. On the one hand, elevated AQP1 expression contributes to increased angiogenesis in astrocytoma, enhancing tumor growth and spread. In human glioblastoma, the disruption of BBB is significantly correlated with the increased AQP4 expression, particularly extended isoform of AQP4 protein (Valente et al., 2022). On the other hand, AQP5 accelerates glioma cell proliferation, migration, and reduces apoptosis via modulation of the EGFR/ERK/p38 MAPK signaling pathway, while AQP8 may also promote astrocytoma proliferation and migration (Zhou et al., 2022), enhancing infiltration into surrounding brain tissue.

Additionally, gliomas alterations the BBB integrity, increased permeability, and impaired endothelial barrier functions further contribute to brain tumor-related epilepsy (Groblewska and Mroczko, 2021). Such as Piezo1, a calcium-permeable transmembrane ion channel protein, is upregulated in glioblastomas. Piezo1 promotes Ca^2+^ influx into vascular endothelial cells, activating calcium-dependent calpains. Calpains further degrade tight junctions between vascular endothelial cells, increasing vascular permeability and exacerbating brain edema (Qu et al., 2020). The relationship between brain tumors and brain edema is complex, driven by alterations in ICP dynamics, vascular changes, and disruptions in the function of essential fluid transport mechanisms.

### 3.5 High-altitude cerebral edema

High-altitude cerebral edema (HACE) is a severe neurological disorder occurring above 2,500 m, affecting millions of residents and visitors annually (Cai et al., 2026). As the end-stage manifestation of acute mountain sickness, HACE is characterized by life-threatening encephalopathy with symptoms including severe headache, ataxia, altered mental status, and impaired cognitive and motor functions (Zhou et al., 2017; Han et al., 2026). Current theory posits that AMS and HACE represent a continuum of neurological dysfunction. Imaging studies have not only confirmed cerebral edema in HACE patients but also suggest that some individuals with AMS may exhibit mild cytotoxic edema in specific brain regions, which could represent an early stage or parallel process to the more severe vasogenic edema observed in HACE (Gatterer et al., 2024).

The primary pathophysiological trigger is hypobaric hypoxia, which initiates a cascade of molecular events leading to cerebral edema (Li et al., 2025). HACE involves vasogenic and cytotoxic edema, driven by BBB disruption via hypoxia-induced upregulation of CAV-1 and degradation of claudin-5 (Wang X. et al., 2022; Xue et al., 2022; Zuo et al., 2025). Furthermore, oxidative stress activates inflammatory pathways through NF-κB and the NOD-like receptor thermal protein domain-associated protein 3 inflammasome. The activated microglia exacerbate BBB damage and contribute to the inflammatory milieu (Wang X. et al., 2022). Concurrently, cytotoxic edema arises from direct cellular dysfunction. Studies reveal that hypoxia induces metabolic disturbances, including glucose metabolic reprogramming and mitochondrial dysfunction, amplifying injury through the opening of the mitochondrial permeability transition pore and the release of apoptotic factors (Han et al., 2024). Single-cell analyses have identified dysregulated oxidative phosphorylation and ribosomal stress in oligodendrocytes and neurons, promoting apoptosis and contributing to edema (Lv et al., 2025). These processes are amplified by a robust neuroinflammatory response mediated by pro-inflammatory cytokines such as TNF-α, IL-1β, and IL-6, and the activation of pathways involving HIF-1α and NF-κB (Li et al., 2025; Zhou et al., 2017; Shi et al., 2020). Thus, HACE pathophysiology is a complex interplay between increased cerebrovascular permeability and direct cellular edema, driven by hypoxia-induced metabolic, inflammatory, and vascular insults.

### 3.6 Anoxic cerebral edema

Anoxic cerebral edema, one of the main causes of neurological disability and mortality, results from severe oxygen deficiency due to events like cardiac arrest or high-altitude exposure (Hanning et al., 2016). The main manifestations include increased ICP, neurological deficits, and, in severe cases, brain herniation. The underlying mechanisms involve a complex interplay leading to both cytotoxic and vasogenic edema. One contributing theory is the “tight-fit” hypothesis, which posits that individuals with less compliant cerebrospinal fluid systems (e.g., smaller ventricles) experience a greater rise in ICP for a given increase in brain volume due to hypoxic swelling (Wilson and Milledge, 2008). While some experimental models suggest that the formation of this type of edema may not initially involve increased permeability of the BBB to large proteins like horseradish peroxidase (Ushijima et al., 1984), other pathways are critically involved.

Recent research has significantly advanced the understanding of the role of neuroinflammation and stress responses in the pathogenesis of anoxic cerebral edema. Systemic pro-inflammatory priming is a key facilitator. Experimental evidence demonstrates that a pre-existing inflammatory state, when combined with acute hypoxia can trigger edema via TLR4/corticotropin-releasing hormone receptor 1 signaling, activating NF-κB/MAPK pathways and upregulating AQP4 expression and increased water permeability in astrocytes (Song et al., 2016). Furthermore, hypoxia itself can elevate plasma levels of pro-inflammatory cytokines (e.g., TNF-α, IL-1β, IL-6) and corticotropin-releasing hormone (Song et al., 2016). Conversely, prophylactic administration of anti-inflammatory and antioxidant agents, such as p-coumaric acid, has been shown to exert protective effects against anoxic cerebral edema in mice. This protection is mediated by reducing oxidative stress, dampening inflammation, enhancing BBBintegrity, and improving Na+-K+-ATPase activity (Li et al., 2019). These findings collectively underscore central roles for inflammatory cascades and cellular stress in driving the molecular and cellular events that culminate in hypoxic brain swelling.

### 3.7 Cerebral edema associated with hydrocephalus

Hydrocephalus-related brain edema coexists with primary CNS injury, and the relationship is not a simple causal one. Instead, they exacerbate each other through two parallel and interwoven pathways: choroid plexus immune activation and compartmentalization disorder of the subarachnoid space. On one hand, intraventricular hemorrhage or pathogen-associated molecular patterns can be recognized by Toll-like receptors on choroid plexus epithelial cells, triggering a nuclear factor kappa B-dependent inflammatory cascade. The upregulation of this pathway directly enhances the expression and phosphorylation of NKCC1, shifting the choroid plexus from ‘secretory homeostasis’ to a hyper-secretory state (Courtney et al., 2025). The increased cerebrospinal fluid production rate elevates intraventricular hydrostatic pressure, which is reversely transmitted to the periventricular white matter spaces, hindering the reflux of interstitial fluid toward the ventricles. This forms the hydrodynamic basis for interstitial edema. Since this type of edema is not accompanied by widespread BBBdisruption, it often appears as smooth periventricular T2 hyperintensity on imaging. On the other hand, the subarachnoid space is not homogeneous. The perivascular subarachnoid space along major arterial trunks is responsible for directional cerebrospinal fluid transport under physiological conditions. In patients with idiopathic normal pressure hydrocephalus, this structure exhibits characteristic phenotypes of enlarged area and prolonged first-pass time of tracers, indicating widened perivascular spaces but significantly reduced anterograde transport efficiency (Eide and Ringstad, 2024). The specific mechanisms—whether due to changes in membrane permeability, weakened vascular pulsation driving force, or compensatory remodeling after chronic inefficient transport—require further investigation.

In hydrocephalus associated cerebral edema, dysfunction of the glymphatic drainage system plays a key role. The glymphatic system is a recently defined brain-wide perivascular network responsible for the exchange between CSF and interstitial fluid and facilitates the clearance of brain metabolic waste (Lv et al., 2021). This system consists of a periarterial CSF influx pathway, convective transport mediated by AQP4 water channels on astrocytic endfeet, and a perivenous efflux pathway, and is closely connected to the meningeal lymphatic system. In various neurological disorders including hydrocephalus, glymphatic system function is disrupted (Al Masri et al., 2024). In hydrocephalus, obstruction of CSF circulation pathways can lead to periventricular interstitial edema, which essentially represents the backflow of CSF into the brain parenchyma through the ependyma (Milhorat, 1992). The glymphatic system, as a core pathway for brain ISF drainage, directly exacerbates such edema when its function is impaired. Studies indicate that in idiopathic normal pressure hydrocephalus, significant glymphatic dysregulation exists, manifested as impaired CSF dynamics and delayed clearance of metabolic waste (Al Masri et al., 2024). Furthermore, in cerebral edema and chronic hydrocephalus following subarachnoid hemorrhage, glymphatic-meningeal lymphatic function is also impaired and is closely associated with elevated extrinsic coagulation pathway factors and inflammatory cytokines (Fang et al., 2024). The AQP4 water channel is a central regulator of glymphatic function (Lv et al., 2021). Its dysfunction is a key link connecting cerebral edema and glymphatic system disruption. In cerebral edema, the functional state of AQP4 determines the direction of water transport: AQP4 deletion aggravates vasogenic edema but alleviates cytotoxic edema. In hydrocephalus, maladaptive changes in AQP4 may further impair glymphatic drainage, and targeting the regulation of AQP4 channels has emerged as a promising therapeutic strategy (Filippidis et al., 2016). For example, in subarachnoid hemorrhage models, the use of tissue plasminogen activator has been shown to alleviate neuroinflammation and cerebral edema, with a potential mechanism possibly involving the restoration of glymphatic-meningeal lymphatic function (Fang et al., 2024).

### 3.8 Hepatic pathology-induced cerebral edema

Hepatic pathology, particularly in the context of acute or chronic liver failure, is a primary instigator of cerebral edema, a severe and often fatal neurological complication. The compromised liver fails to adequately detoxify the bloodstream, leading to the systemic accumulation of neurotoxic substances (Kaler, 2016). This toxic milieu is further exacerbated by systemic inflammatory responses originating from the injured liver; for instance, conditions like metabolic dysfunction-associated steatohepatitis are characterized by the release of pro-inflammatory mediators such as periostin, which can disseminate via circulation and potentially impact distant organs including the brain (Xie et al., 2024). Furthermore, the gut-liver axis plays a critical role, as hepatic dysfunction alters bile acid metabolism and compromises intestinal barrier integrity, facilitating the translocation of bacterial products like lipopolysaccharide into the portal circulation (Barretto et al., 2021; Luo et al., 2025). These circulating toxins and inflammatory signals collectively assault the CNS, setting the stage for edema formation.

The liver’s inability to clear ammonia leads to hyperammonemia, which disrupts cerebral amino acid metabolism and osmotic regulation within astrocytes (Ajoolabady et al., 2023). Concurrently, alterations in aromatic amino acid metabolism contribute to the synthesis of false neurotransmitters, disrupting normal neuronal function. These metabolic insults converge to destabilize the BBB and induce astrocyte swelling, the hallmark of cytotoxic edema (Ibrahim et al., 2018). At a cellular level, similar pathogenic processes observed in hepatic cells, such as endoplasmic reticulum stress and iron overload-induced ferroptosis, may have parallels in neural cells, promoting oxidative stress and cell death that exacerbate edema (Guo J. et al., 2021; Ajoolabady et al., 2023; Liu et al., 2025). The activation of stress-response pathways like KEAP1-NRF2 in hepatocytes under pathological conditions suggests a systemic redox imbalance that could similarly affect the vulnerable brain parenchyma (Liang et al., 2026).

Severe hepatic pathologies, including advanced cirrhosis or acute failure, can lead to cardiopulmonary complications and hemodynamic instability, resulting in cerebral hypoperfusion and hypoxia (Malka-Markovitz et al., 2025). This hypoxia disrupts cellular energy metabolism, leading to ionic pump failure, intracellular sodium and water accumulation, and subsequent swelling of neuronal and glial cells. Moreover, intravital imaging studies in steatohepatitis models reveal a dynamic, pathological microenvironment with immune cell activation and cluster formation, which in the brain could correlate with neuroinflammatory responses that worsen hypoxic injury and edema (Wang et al., 2021). The interplay between systemic inflammation from liver disease, endothelial dysfunction, and reduced oxygen delivery creates a vicious cycle that amplifies hypoxic brain damage.

### 3.9 Renal pathology-induced cerebral edema

Renal pathology can significantly influence cerebral edema through systemic metabolic disturbances. Severe kidney disease, particularly acute or chronic renal failure, impairs the body’s ability to excrete metabolic waste products and maintain electrolyte and acid–base homeostasis. This results in the accumulation of uremic toxins, hyperkalemia, and metabolic acidosis. These systemic alterations compromise CNS function, leading to cellular swelling.

The mechanisms by which renal pathology leads to cerebral edema are multifaceted, involving direct metabolic pathways and secondary inflammatory responses. Firstly, kidney failure causes profound metabolic and excretory changes. The retention of urea and other nitrogenous wastes contributes to osmotic imbalances. Similar to how hepatic dysfunction alters metabolite profiles (Guo J. et al., 2021), renal failure disrupts the systemic balance of solutes, creating an osmotic gradient that favors water movement into brain cells. Furthermore, impaired renal excretion leads to the accumulation of cytokines and inflammatory mediators, fostering a state of systemic inflammation. This inflammation is a key driver of endothelial dysfunction, as observed in various hepatic pathologies (Xie et al., 2024; Zhang H. et al., 2024). Systemic inflammation can weaken the BBB, a critical finding in studies of other organ-related encephalopathies (Lagana et al., 2020).

Secondly, the direct pathway to cerebral edema involves BBB disruption and cellular swelling. Uremic toxins and inflammatory cytokines can directly activate endothelial cells and pericytes, compromising tight junction integrity. Subsequently, astrocytes, which play a crucial role in brain ion and water homeostasis, undergo swelling. This systemic disturbances trigger neuroinflammation and oxidative stress, leading to glial cell injury and swelling (Ajoolabady et al., 2023; Wang C. Y. et al., 2022). Thus, renal failure creates a cascade from systemic solute imbalance and inflammation to direct BBB injury and cellular edema in the brain.

## 4 Neurological regulatory mechanisms

The brain employs several regulatory mechanisms to maintain homeostasis and protect against edema. These mechanisms involve the coordination of various cellular and molecular processes.

### 4.1 Blood–brain barrier

The BBB is a crucial anatomical and physiological interface that protects the CNS from potentially harmful substances while allowing essential nutrients to pass through. It is primarily composed of specialized endothelial cells, astrocytes, and pericytes, forming a selective barrier that regulates the movement of molecules between the blood and the CNS (Figure 2Aa). The integrity of the BBB is primarily maintained by tight junctions between endothelial cells, composed of transmembrane proteins including claudins and occludin, and anchored to the cytoskeletal framework via zonula occludins proteins. Under normal physiological conditions, the BBB effectively restricts the permeability to large molecules and ions, thus preventing the accumulation of excess fluid and maintaining the delicate balance required for optimal neuronal function (Keep et al., 2018). The integrity of the BBB is vital for preventing cerebral edema, which can occur when the barrier is compromised, leading to increased permeability and subsequent fluid accumulation in the brain parenchyma. Understanding the structural components and functional roles of the BBB is essential for elucidating the mechanisms underlying various neurological disorders, including TBI, stroke, and neuroinflammatory conditions (Le Guennec and Weiss, 2023).

In pathological states such as TBI and inflammation, the integrity of the BBB can be severely compromised. Mechanistically, this is often associated with a reduction in the expression of tight junction proteins, such as occludin and claudin-5, which are critical for maintaining the barrier’s selective permeability (Gao and Hernandes, 2021). Inflammatory mediators, including tumor necrosis factor-alpha (TNF-α) and interleukin-1 beta (IL-1β), can exacerbate this permeability by triggering signaling cascades that lead to the disruption of endothelial cell junctions and the activation of MMPs, which degrade the extracellular matrix components supporting the BBB (Wang et al., 2023; Versele et al., 2022). Additionally, the generation of reactive oxygen species during inflammatory responses can further damage endothelial cells, contributing to increased BBB permeability. The resultant breakdown of the BBB allows for the extravasation of plasma proteins and fluids into the brain interstitium, leading to vasogenic edema—a condition characterized by the accumulation of fluid in the extracellular space, which can significantly impair neurological function and increase ICP (Gan et al., 2024). Therefore, targeting the inflammatory mediators and signaling pathways involved in BBB disruption presents a potential therapeutic strategy to mitigate cerebral edema and its associated complications (Lu et al., 2024).

The repair of the BBB following injury is a complex process that involves various signaling pathways and molecular mediators. VEGF and MMP play dual roles in both the damage and repair of the BBB. While VEGF is known to promote endothelial cell proliferation and migration, excessive levels can also lead to increased permeability and edema (Bernocchi et al., 2024; Matsuno et al., 2022). Recent studies have highlighted the importance of the Wnt/β-catenin signaling pathway in regulating BBB integrity, with activation of this pathway contributing to the stabilization of tight junctions and the restoration of barrier function following injury (Shawahna et al., 2017). Transforming growth factor-beta (TGF-β) is another critical player that modulates the inflammatory response and promotes the expression of tight junction proteins, thus aiding in the repair of the BBB (Zheng et al., 2025). Furthermore, research indicates that pharmacological modulation of these signaling pathways may offer therapeutic benefits in reducing BBB dysfunction and subsequent cerebral edema. For instance, targeting the Wnt/β-catenin pathway has shown promise in experimental models, suggesting that enhancing this signaling could mitigate BBB breakdown and improve outcomes in conditions associated with cerebral edema (Shawahna et al., 2017). Overall, the BBB plays a crucial role in the development and regulation of brain edema. Understanding the complex interactions that govern BBB integrity and function remains essential for the development of effective interventions in various neurological disorders characterized by edema.

### 4.2 Glial plasticity

Glial cells, which include astrocytes, oligodendrocytes, and microglia, play fundamental roles in the central CNS, contributing to various physiological and pathological processes.

Astrocytes are the most abundant type of glial cell in the CNS. As Figure 2A shows, the processes of astrocytes directly attach to the endothelial cells of capillaries in the brain, making them an important component of the BBB. Astrocytes secrete brain-derived neurotrophic factor (BDNF) (Albini et al., 2023) and nerve growth factor, which effectively promote the survival, growth, and differentiation of neurons. On the other hand, they are also closely associated with the axon terminals at synaptic connections, helping to reduce the cytotoxicity to the CNS by recycling excess excitatory neurotransmitters such as glutamate (de Ceglia et al., 2023). Astrocytes take up glutamate, stimulating their anaerobic glycolysis to convert glucose into lactate, which is then transmitted through axons to neurons, serving as a metabolic substrate to provide energy for neurons. Additionally, Astrocytes secrete VEGF to maintain the survival of endothelial cells and the stability of blood vessels (Tabata, 2022). In the early stages of brain edema, the secretion of VEGF and MMPs by glial cells can lead to the degradation of tight junction proteins between endothelial cells, resulting in increased BBB permeability and subsequent edema formation (Hernandes et al., 2018). Following CNS injury, astrocytes are activated, leading to the upregulation of inflammatory mediators such as cytokines and chemokines. This activation is marked by the expression of glial fibrillary acidic protein (GFAP), a key indicator of astrocyte reactivity (Li et al., 2020). The sustained release of pro-inflammatory cytokines can disrupt the BBB, leading to increased vascular permeability and subsequent fluid accumulation in the brain tissue (Yang et al., 2022). Conversely, astrocytes regulate ion homeostasis and water transport through the expression of AQPs by GFAP, which causes the occurrence of cytotoxic edema (Li et al., 2020; Guo H. et al., 2021). The retraction of astrocyte processes reduces the physical barrier between AVP neurons, leading to increased activation of these neurons and secretion of AVP, thereby exacerbating brain edema. In the late stages, astrocytes contribute to the partial restoration of BBB integrity by secreting MMP inhibitors and growth factors that promote vascular repair and stability (Du et al., 2025). On the other hand, astrocytes proliferate and form a glial scar, which serves to limit the spread of injury but paradoxically may impede neural regeneration and repair processes (Catalin et al., 2018; He et al., 2022). AQP4 expression can be further upregulated or redistributed in response to edema, impacting the transmembrane transport of water and contributing to the severity of edema (Ishikawa and Yasui, 2021). Astrocytes resecrete BDNF, which activates neuronal TrkB receptors to promote neuronal survival (Li C. et al., 2021b). Astrocytes secrete VEGF and basic fibroblast growth factor (bFGF) to promote endothelial cell proliferation (Deng et al., 2020). Simultaneously, they secrete TGF-β to regulate the expression of tight junction proteins in new vascular endothelial cells, promoting partial recovery of BBB function (Tabata, 2022). During the recovery phase, astrocytes restore the normal expression level ofAQP4, promoting the clearance of excess extracellular fluid to restore body fluid balance (Stokum et al., 2015). Understanding these complex interactions is vital for developing targeted therapies on cerebral edema.

Microglia are the resident immune cells of the CNS, playing a pivotal role in maintaining brain homeostasis and responding to injury and disease. Microglia participate in the regulation of the BBB structure and function through various mechanisms (Mayer and Fischer, 2024). For example, in the adult brain, the loss of microglia-derived platelet-derived growth factor-B leads to BBB disruption in adult mice (Weng et al., 2024). Additionally, microglia also play a crucial role in strengthening the physical barrier functions of the BBB by influencing the expression of tight junction proteins in endothelial cells (Zhang S. et al., 2024). Notably, microglia exhibit a dual role during inflammation; while they may contribute to the development of edema in the early phase, they can also promote tissue repair and protection in the later stage (Yang J. et al., 2021). Upon activation due to ischemic stroke or intracerebral hemorrhage, microglia can polarize into pro-inflammatory (M1) microglia, which release pro-inflammatory cytokines, such as TNF-α and IL-1β, which aggravates both BBB disruption and subsequent edema in TBI, stroke and hypoxia (Wang X. et al., 2022). Meanwhile, the generation of ROS is also significantly increased during microglial activation, further exacerbating neuroinflammation and oxidative stress damage (Huang et al., 2024). Specifically, studies have shown that in LPS-treated BV-2 microglial cells, ROS levels rise while the activity of antioxidant enzymes such as glutathione peroxidase decreases (Jin J. et al., 2020). Additionally, in animal models of intracerebral hemorrhage, the M1 polarization of microglia is closely associated with the release of pro-inflammatory cytokines, which can be alleviated by modulating specific signaling pathways such as TLR4/NF-κB. Furthermore, the activation of microglia is influenced by Toll-like receptors and nuclear factor kappa-light-chain-enhancer of activated B cells, which mediate the inflammatory response and brain edema (Deng et al., 2023). In the later stage, anti-inflammatory (M2) microglia release neurotrophic factors and cytokines that promote neuronal survival and repair (Figure 2B; Zhang Z. et al., 2022). Such as, IL-4 in the hypoxic ischemia brain site induces the M2 polarization of microglia, releasing BDNF, NGF-1 to protect BBB integrity and neuronal survival (Zhou et al., 2020). Furthermore, microglial exosomes have been implicated in mediating neuroprotective effects and promoting recovery after injury (Li C. et al., 2021a). In diseases, microglia also communicate with astrocytes through various signaling pathways to influence their activation states and the overall inflammatory response in the brain (Rostami et al., 2021). Understanding these cellular interactions paves the way for innovative therapeutic strategies that focus on improving neuroprotection and facilitating recovery after CNS injuries.

Oligodendrocytes are specialized glial cells in the CNS, primarily responsible for the formation and maintenance of myelin sheaths that insulate axons, facilitating rapid electrical signal conduction. Oligodendrocytes not only support neuronal function but also help to regulate the ionic milieu essential for neuronal excitability and signaling. Inward rectifying potassium channels are prominently expressed in oligodendrocytes, facilitating potassium uptake and contributing to the maintenance of the resting membrane potential (Papanikolaou et al., 2020). Furthermore, oligodendrocytes respond to signals from activated microglia by releasing cytokines, which can either support the survival of oligodendrocytes or contribute to their degeneration, ultimately influencing neuronal activity (Chen et al., 2023). Thus, oligodendrocytes are not merely passive insulators but active participants in CNS signaling and homeostasis.

### 4.3 Cerebral blood flow autoregulation

CBF autoregulation is the process of regulating the constriction or dilation of cerebral blood vessels by altering the difference between mean arterial pressure and ICP, known as cerebral perfusion pressure (Kaur and Sharma, 2018). Autoregulation operates through a complex interplay of myogenic responses, neurotransmitter interactions, and local metabolic demands. For instance, the primary mechanism of myogenic autoregulation focuses on modifying cerebral vascular resistance through vasoconstriction and vasodilation in response to fluctuations in cerebral perfusion pressure. When autoregulation is fully functional, CBF can be preserved as cerebral perfusion pressure declines toward the lower threshold of autoregulatory vasodilation. However, if cerebral perfusion pressure drops below this autoregulatory threshold, it triggers maximal vasodilation of the blood vessels, and physiological autoregulatory vasodilation or hyperemia may result in elevated ICP, thereby worsening brain edema (Kofke et al., 2020; Zhang X. et al., 2022).

The brain’s ability to autonomously regulate blood flow is closely related to the integrity of the BBB (Barkaway et al., 2022; Li S. et al., 2023). Disruption of the BBB can exacerbate the formation of cerebral edema. For instance, in stroke models, bone marrow stromal cell transplantation has been shown to restore CBF and BBB function in a dose-dependent manner, as verified by laser Doppler flowmetry and Evans Blue assays, indicating that CBF and BBB return to normal levels at an earlier stage after transplantation (Borlongan et al., 2004). Additionally, pericytes, which are contractile cells located on the surface of capillaries, play a key role in regulating capillary tension and diameter, thereby influencing local blood flow. Pericytes maintain BBB integrity by inhibiting endothelial cell transcytosis and inducing the expression of tight junction proteins. However, their loss or dysfunction may lead to BBB leakage and impaired CBF (Barkaway et al., 2022; Wang Y. et al., 2022).

Under pathological conditions, such as placental ischemia or infection, autoregulation and BBB function may be further compromised. For example, in a rat model of placental ischemia, impaired CBF autoregulation and increased BBB permeability led to forebrain edema, which was associated with changes in the expression of tight junction proteins such as claudin-1 (Warrington et al., 2014). Similarly, Toxoplasma gondii infection can trigger the upregulation of adhesion molecules, such as ICAM-1 and VCAM-1, in brain endothelial cells, resulting in vascular occlusion and reduced CBF (Hoover et al., 2025). Ultrasound-mediated BBB opening has also been shown to cause reduced CBF and increased arterial transit time, indicating that BBB disruption is associated with CBF hypoperfusion (Labriji et al., 2023). One critical aspect of CBF autoregulation is the expression of membrane proteins such as Mfsd2a, a major facilitator superfamily domain-containing protein 2A. During the acute phase of TBI, the expression of Mfsd2a is downregulated, leading to a compromised BBB, exacerbating edema formation (Zhang Y. et al., 2022). In summary, CBF autoregulation and BBB integrity are interdependent, and various factors, such as pericyte function, inflammatory responses, and vascular changes, can influence this balance, leading to adverse outcomes in brain injury or disease.

### 4.4 Cellular ion homeostasis

The stability of cellular ion concentrations is essential for the normal structure and function of neurons and glial cells. When this balance is disrupted, the buildup of electrolytes inside the cells creates an osmotic pressure gradient, which raises the osmotic pressure in brain tissue. This condition allows blood from the cerebral blood vessels to permeate into the brain tissue, ultimately resulting in cytotoxic edema (Biller et al., 2021). In cytotoxic edema, astrocytes immediately swell, which affects the Na^+^-K^+^-ATPase, worsening the imbalance of sodium and potassium ions both inside and outside the cells (van Putten et al., 2021). The activation of Na^+^-K^+^-ATPase drives NKCC1 in both astrocytes and endothelial cells promoting the development of brain edema (Hertz et al., 2017). Similarly, in the cerebral ischemic stroke, the blockage of cerebral vessels causes rapid oxygen and glucose deprivation, halting ATP synthesis and inactivating the ATP-dependent Na+-K+-ATPase. This accelerates the development of brain edema and results in irreversible neuronal injury or death (Kofke et al., 2020; Hellas and Andrew, 2021). Thus, the dysregulation of Na^+^ and K^+^ ions is a pivotal mechanism underlying the initiation and progression of brain edema. Previous studies have indicated that blocking voltage-gated sodium channels may exert neuroprotective effects in ischemia-induced brain seizures (Williams and Tortella, 2002). On the contrary, inhibiting the sodium/calcium exchangers can lead to osmotic cell death in stroke (Stokum et al., 2023). In ischemic stroke, the efflux of K^+^ from astrocytes exacerbates the accumulation of extracellular K^+^, which activates NKCC1 and facilitated the influx of sodium ions (van Putten et al., 2021). Late-stage changes frequently involve the disruption of the blood–brain barrier, which triggers an inflammatory response that results in vasogenic cerebral edema. To sum up, the regulation of Na^+^ and K^+^ plays a crucial role in the pathophysiology of brain edema.

Besides cations, anions also have a crucial role in brain edema. For example, chloride ions, which are the most prevalent anions in the human body and are key contributors to cell swelling and the regulation of volume. The chloride channel family consists of voltage-gated Cl^−^ channels and H^+^/Cl^−^ exchangers. The voltage-gated Cl^−^ channels are predominantly found in astrocytes, oligodendrocytes, pyramidal and non-pyramidal neurons in the brain. When ATP is depleted, these channels remain in an open state. Voltage-gated Cl^−^ channels alleviate intracellular cation accumulation caused by K^+^ or Na^+^ channels in glial cell edema (Pasantes-Morales and Vazquez-Juarez, 2012). Additionally, the chloride channel-3 aids in alleviating further damage in cerebral ischemia by promoting autophagic activation (Zhang B. et al., 2020). Research has found that the activation of proton-activated Cl^−^ under acidic pathological conditions leads to chloride ion influx and cellular edema. However, the knockout of the proton-activated Cl^−^ gene in mice with permanent middle cerebral artery occlusion resulted in significant improvements in brain injury and neurological function (Yang et al., 2019). The inhibition of sulfonylurea receptor 1 can thereby suppress the formation of brain edema through NCCa-ATP channel (Alquisiras-Burgos et al., 2020).

### 4.5 Arginine-vasopressin regulation

AVP, also known as antidiuretic hormone, was discovered by Oliver and Schafer. The discovery was prompted by an observation of remarkable elevation of blood pressure in individuals who received intravenous injection of pituitary extract (Oliver and Schafer, 1895). AVP regulates water balance, blood pressure, platelet function, and thermoregulation (Atila et al., 2024). The precursor of AVP is synthesized in magnocellular neurons in the supraoptic and paraventricular nuclei of the hypothalamus. AVP is transported to the axon terminals of magnocellular neurons in the posterior pituitary by binding to carrier protein neurophysin.

The main physiological mechanisms controlling the release of AVP are osmotic stimulation, changes in blood volume, and pressure. However, the secretion of AVP also occurs in response to stress-related stimuli. In stroke, brain edema directly compresses the hypothalamus, leading to the release of AVP from the paraventricular nucleus and the supraoptic nucleus. Additionally, the release of glutamate, the formation of a local hyperosmotic environment, changes in the structure and function of astrocytes, and the release of pro-inflammatory mediators exacerbate the secretion of AVP (Chojnowski et al., 2023).

It primarily exerts its effects through three receptor subtypes: V1a, V1b, and V2. The V1a and V2 receptors are particularly significant in mediating vasoconstriction and increasing vascular permeability. Studies have shown that AVP can exacerbate conditions such as brain edema by increasing the permeability of the BBB and promoting fluid accumulation in the brain tissue. This effect is particularly pronounced in pathological states such as TBI and subarachnoid hemorrhage, where the overactivity of AVP can lead to exacerbated brain edema (Tan et al., 2019). AVP receptor antagonists can mitigate the effects of AVP on vascular permeability, thereby reducing brain edema in intracerebral hemorrhage (Vinuela-Berni et al., 2020). Furthermore, AVP involvement in the inflammatory cascade highlights the dual functional role. It modulates the secretion of pro-inflammatory cytokines such as IL-6 and TNF-α (Yang et al., 2020; Fan et al., 2024) through V1a receptor, leading to a cascade of events that promote edema and neuronal damage (Ohmura et al., 2023). Conversely, AVP also exhibits anti-inflammatory properties in certain contexts. Research indicates that AVP can modulate the inflammatory response by influencing the activity of immune cells and the release of anti-inflammatory cytokines (Ohmura et al., 2023; Kato et al., 2024). For instance, AVP has been shown to inhibit the activation of microglia (Vinuela-Berni et al., 2020). The dual role of AVP—acting as both a pro-inflammatory and an anti-inflammatory mediator—highlights the complexity of its actions in the context of cerebral edema.

## 5 Signal passengers

### 5.1 Effects of nitric oxide on brain edema

Nitric oxide (NO) is a chemically active gas that serves both intracellular and intercellular signaling roles as a messenger and neurotransmitter. The synthesis of NO is achieved by promoting the enzymatic conversion of L-arginine to NO and L-citrulline through nitric oxide synthase (NOS). The brain tissue is also a site of endogenous NO synthesis and was the first to isolate and purify NOS from the rat cerebellum (Feng et al., 2025). Subsequently, Rodrigo et al. (1994) conducted immunohistochemical localization studies of NOS distribution in the rat brain using NOS antisera, finding NOS in various areas such as the granule cell layer of the cerebellum, the olfactory bulb, the hippocampus, and the dentate gyrus. There are three main subtypes of NOS: neuronal type (nNOS), endothelial type (eNOS), and inducible type (iNOS).

In the CNS, nNOS is primarily located in neuronal terminals, promotes NO release, and modulates neurotransmitter release and synaptic function to maintain neuronal homeostasis (Pitsikas, 2024). eNOS plays a crucial role by producing NO with its activation being regulated by calmodulin. When eNOS is activated, it leads to an increase in cyclic guanosine monophosphate within vascular smooth muscle cells. This increase causes the dephosphorylation of myosin light chains. This biochemical cascade effectively inhibits vasoconstriction, promotes vascular relaxation, and regulates local blood flow, thereby helping to maintain the integrity and normal permeability of the BBB (Gericke and Buonfiglio, 2024). Consequently, during the early stages of conditions like cerebral ischemia, NO helps maintain CBF, reduce oxidative stress, and preserve the integrity of the BBB. For example, adropin treatment upregulates eNOS phosphorylation through an eNOS-dependent mechanism, alleviates BBB damage and neuronal apoptosis, thereby improving early ischemic injury (Yang C. et al., 2021). eNOS drives astrocyte VEGF to promote BBB integrity (Lopez-Ramirez et al., 2021). Similarly, induced pluripotent stem cell-derived small extracellular vesicles activate the eNOS-Sirt1 axis, enhance BBB function, and inhibit inflammatory responses (Li Q. et al., 2023).

As the disease advances to its later stages, iNOS is overactivated, leading to the production of a large amount of NO, which triggers oxidative stress, inflammation, and cytotoxicity, exacerbating brain damage. For example, neutrophils express iNOS in the area of cerebral infarction (Iadecola et al., 1995), the overexpression of iNOS is associated with increases in malondialdehyde, advanced glycation end products, and pro-inflammatory cytokines (such as TNF-α and IL-1β). These factors together promote BBB disruption and neuronal death (Barakat et al., 2018). Meanwhile, the expression of eNOS may be reduced or its function may be impaired, further weakening the protective effect; partial eNOS deficiency can spontaneously cause thrombosis, BBB breakdown, and cognitive impairment (Tan et al., 2015). Overall, the imbalance in the role of NO (weakened protective effect of eNOS and enhanced destructive effect of iNOS) can lead to pathological changes such as BBB damage and cerebral edema.

### 5.2 The role of glutamate in brain edema

Glutamate is the main excitatory neurotransmitter in the CNS, present in almost all areas of the brain. Its receptors are widely distributed and expressed on both neuronal and non-neuronal cells. Glutamate is vital in the development of brain edema, through mechanism that includes increased permeability of the BBB, swelling of astrocytes, and changes in cellular water balance. Research indicates that glutamate levels in the cerebrospinal fluid rise significantly, correlating with the severity of brain edema and neurological impairments following subarachnoid hemorrhage (Zhang C. et al., 2020). Furthermore, elevated glutamate concentrations can disrupt the integrity of tight junctions between endothelial cells, worsening the breakdown of the BBB (Yemanyi et al., 2021). The deposition of glutamate around cerebral edema leads to the opening of NKCC1, promoting the influx of sodium ions into brain cells and exacerbating cytotoxic edema (Chen Y. et al., 2021). At the same time, astrocytes can uptake glutamate through excitatory amino acid transporters 1 and 2, which facilitates the transport of sodium ions. This process can carry over 400 water molecules and is accompanied by the reverse transport of one K^+^ ion. Furthermore, glutamate activates its metabotropic receptors, inducing membrane depolarization in astrocytes, which activates Na^+^/K^+^-ATPase and results in swelling of both brain cells and astrocytes (Zhou et al., 2022). Additionally, glutamate binds to metabotropic glutamate receptors and accumulates in endothelial cells, driving excitotoxicity and cell death, leading to the swelling of astrocytes (Shi et al., 2017). These findings underscore the critical role of glutamate in the pathophysiology of brain edema and highlight the potential for therapeutic strategies aimed at modulating glutamate signaling to prevent or reduce edema formation in various neurological conditions.

### 5.3 Other signaling molecules involved in edema regulation

Signaling molecules play a crucial role in the development of brain edema. For instance, Sphingosine-1-phosphate strengthens tight junctions in endothelial cells by binding to its receptors, which helps protect the BBB from damage (Ziegler and Graler, 2021). On the other hand, inflammatory agents like TNF-α and IL-1β can compromise the BBB and induce brain edema by activating specific signaling pathways (Wang X. et al., 2022). In models of acute ischemic stroke, inhibiting p38 MAPK has been shown to significantly reduce inflammation, thereby mitigating brain injury and edema (Chen et al., 2024). Additionally, signaling pathways such as TGF-β and nuclear factor kappa B contribute to the expression of inflammatory factors and the activation of cells, further influencing the development of brain edema. Recent studies have highlighted the important roles of novel signaling molecules in this process. For example, the p38 mitogen-activated protein kinase pathway is implicated in brain edema induced by 1,2-dichloroethane in mice, as it promotes the overexpression of MMP-9 and activates nuclear factor kappa B. Furthermore, in cerebral hemorrhage models, inhibiting the sonic hedgehog pathway in astrocytes can impair the maturation and integrity of the BBB, worsening brain edema and neurological deficits (Xing et al., 2020). Future research should focus on elucidating the roles of these signaling molecules and exploring their potential clinical applications, with the goal of developing new strategies for the prevention and treatment of brain edema.

## 6 Clinical implications

### 6.1 Pharmacological treatment

**Figure 3:**
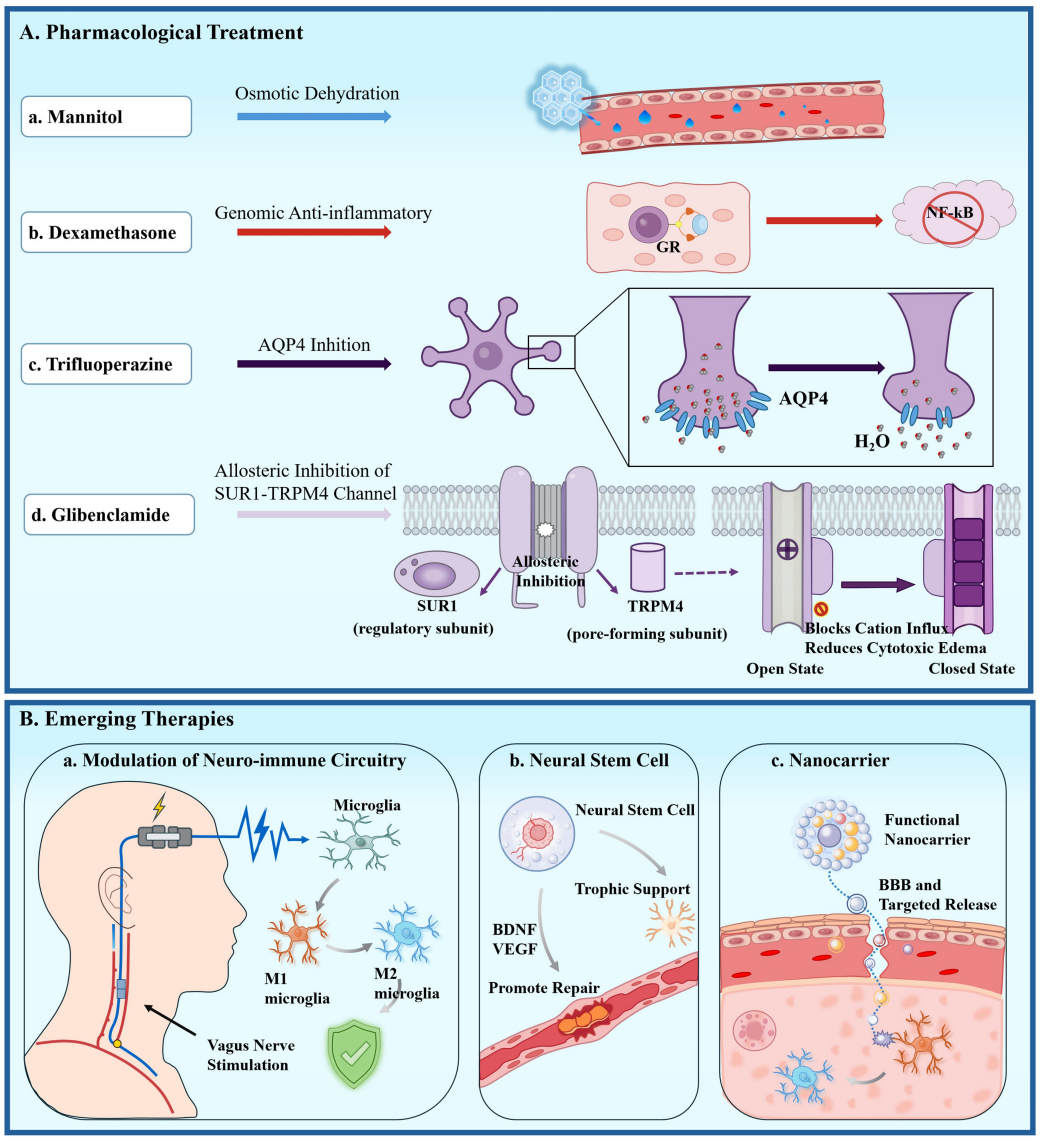
The schematic systematically illustrates the potential therapeutic mechanisms for cerebral edema. **(A)** Pharmacological treatment. **(a)** After intravenous infusion of mannitol, a hypertonic environment is established in the blood vessels, forming an osmotic pressure gradient across the blood–brain barrier, which reversely draws water molecules from the interstitial fluid of the brain tissue into the vascular lumen; **(b)** dexamethasone activates intracellular glucocorticoid receptors, inhibits the transcriptional activity of pro-inflammatory signaling pathways such as NF-κB, and downregulates the expression of pro-inflammatory cytokines, thereby alleviating inflammatory responses and vascular permeability; **(c)** inhibition of the AQP4 on astrocytic endfeet effectively reduces water permeation into brain tissue; **(d)** glibenclamide allosterically inhibits the SUR1-TRPM4 channel complex. Upon binding to the SUR1 regulatory subunit, it induces closure of the TRPM4 pore-forming subunit, blocks the influx of cations such as Na^+^ and Ca^2+^, and thereby reduces cellular swelling and cytotoxic edema. **(B)** Emerging therapies. **(a)** Neuromodulation regulates microglial phenotype; **(b)** stem cell therapy exerts dual neuroprotective and vasculotropic effects via paracrine signaling; **(c)** targeted nanocarriers deliver therapeutics to diseased immune cells and endothelial cells. NF-κB, nuclear factor kappa-light-chain-enhancer of activated B cells; GR, glucocorticoid receptor; AQP4, aquaporin 4; SUR1-TRPM4, sulfonylurea receptor 1-transient receptor potential melastatin 4; BDNF, brain-derived neurotrophic factor; VEGF, vascular endothelial growth factor.

The management of cerebral edema primarily relies on diuretics and anti-inflammatory medications to reduce excess fluid in the brain and address related physiological changes (Figure 3A). Mannitol is one of the most commonly used diuretics in clinical settings; it acts as an osmotic agent that pulls water out of brain tissue and into the bloodstream, effectively lowering ICP. This effect occurs because mannitol creates an osmotic gradient that facilitates the movement of water from both intracellular and interstitial spaces into the vascular system, thereby decreasing cellular swelling and alleviating edema (Koenig, 2018). Additionally, mannitol has been shown to have neuroprotective effects, including the preservation of BBB integrity and the reduction of free radical formation, which further contributes to its therapeutic benefits in conditions associated with cerebral edema, such as TBI and stroke (Qiao et al., 2025). In addition to diuretics, anti-inflammatory medications, particularly corticosteroids, are used to manage cerebral edema, especially in cases related to tumors or post-surgery. Dexamethasone, a strong corticosteroid, is frequently preferred due to its effectiveness in reducing inflammation and vascular permeability, which helps to lessen edema (Yang S. et al., 2021). However, the long-term use of corticosteroids is associated with significant side effects, including immunosuppression and metabolic disturbances, which necessitate careful consideration of their use in clinical settings (Doumeizel et al., 2021).

Emerging research has also highlighted the role of AQP4 that facilitates water transport across cell membranes, and its dysregulation has been implicated in various neurological conditions. Pharmacological agents that target AQP4, such as trifluoperazine, have shown promise in preclinical studies by reducing edema and improving neurological outcomes following TBI (Sylvain et al., 2021). The inhibition of AQP4 may help restore normal water homeostasis in the brain, thus providing a novel therapeutic avenue for the treatment of cerebral edema. Furthermore, newer agents like glyburide, which inhibit the sulfonylurea receptor 1-transient receptor potential melastatin 4 ion channel, have emerged as promising therapeutic targets for managing cerebral edema (King et al., 2018). The advancement of nanodrug delivery systems that can cross the BBB and specifically target certain cell types in the brain may further improve the effectiveness of pharmacological treatments for cerebral edema (Fu et al., 2025).

In summary, while current treatments for cerebral edema primarily involve diuretics and anti-inflammatory medications, ongoing research into the roles of AQPs and ion channels offers exciting possibilities for developing more effective and targeted therapies. Integrating these innovative approaches into clinical practice could significantly enhance patient outcomes in conditions associated with cerebral edema.

### 6.2 Emerging therapies

Emerging therapies for cerebral edema, particularly in the context of neurological injuries and disorders, are gaining traction due to their potential to address the underlying pathophysiological mechanisms rather than merely alleviating symptoms. As Figure 3B shows, neuroregulatory techniques and stem cell therapies are leading the way. For example, vagus nerve stimulation (VNS) has shown potential in modulating neuroinflammation and enhancing outcomes in conditions like ischemic stroke, where cerebral edema poses a significant challenge. VNS has been associated with reduced edema and improved BBB integrity, suggesting a multifaceted approach to treating cerebral edema by addressing both inflammation and vascular permeability (Weiss and Ding, 2025). Additionally, the inhibition of edema-related molecules, such as AQP4 and sulfonylurea receptor 1, represents a targeted strategy to mitigate edema formation. Research has shown that SUR1 inhibitors can effectively prevent edema in preclinical models, suggesting that these molecular targets may soon be applicable in clinical settings (Jha et al., 2021).

Stem cell therapy is another innovative approach that has garnered attention for its regenerative potential in treating cerebral edema. Research has highlighted the efficacy of human neural stem/progenitor cells in reducing edema and promoting recovery following TBI. In studies using rat models, the transplantation of these cells, when combined with curcumin-loaded niosome nanoparticles, led to significant improvements in locomotor activity, as well as reductions in brain edema, gliosis, and inflammatory responses (Narouiepour et al., 2022). This combination therapy appears to harness the protective and regenerative properties of stem cells while simultaneously addressing the inflammatory milieu that exacerbates edema. Furthermore, the application of gene therapies and recombinant proteins is being explored to further enhance the therapeutic landscape for cerebral edema. These emerging modalities may provide new avenues for intervention, particularly in cases where traditional treatments have failed or are insufficient.

The potential of these emerging therapies lies not only in their ability to reduce cerebral edema but also in their capacity to repair and regenerate neural tissue. As our understanding of the molecular and cellular mechanisms underlying cerebral edema deepens, the development of targeted therapies that can effectively restore BBB integrity and modulate inflammatory responses will likely become integral to the management of conditions associated with cerebral edema. Ongoing clinical trials and research will be vital in assessing the safety, effectiveness, and best applications of these innovative strategies in real-world scenarios, ultimately striving to enhance patient outcomes and alleviate the impact of cerebral edema in neurological disorders (Pico et al., 2024).

## 7 Conclusion

The mechanisms behind cerebral edema are complex and involve a variety of neurobiological factors working together. This review emphasizes the significant impact of BBB disruption, inflammatory responses, and intracellular edema on the development of cerebral edema. A deeper understanding of these processes not only elucidates the pathophysiology of cerebral edema but also unveils novel avenues for clinical treatment strategies, potentially improving patient outcomes. Additionally, examining the mechanisms of intracellular edema adds further complexity to our knowledge of cerebral edema. Recent studies suggest that cellular swelling can occur due to several factors, such as ionic imbalances and metabolic disturbances. Therefore, future research should aim to clarify these pathways, as targeting intracellular mechanisms could lead to innovative therapeutic options (Table 2).

**Table 2:** Target molecules of brain edema.

Category	Target molecules	Mechanism of action
Inflammatory mediators	TNF-α	Disrupts tight junctions of the blood–brain barrier, increasing permeability and leading to vasogenic edema
	IL-1β	Triggers signaling cascades that disrupt endothelial cell junctions, exacerbating BBB permeability
	TLR4	Mediates persistent inflammation leading to BBB disruption, brain edema, etc.; its inhibition shows therapeutic potential
Signaling pathway molecules	MMP-9	Degrades the extracellular matrix, compromising BBB integrity; a potential target for therapeutic intervention
	p38 MAPK	Promotes inflammation and MMP-9 overexpression, exacerbating brain injury and edema; inhibition of p38 MAPK can alleviate edema
	NF-κB	Involved in the expression of inflammatory factors and cell activation, influencing the development of brain edema
	Wnt/β-catenin	Stabilizes tight junctions and promotes the restoration of BBB function after injury; activation of this pathway may mitigate brain edema
	TGF-β	Modulates the inflammatory response and promotes the expression of tight junction proteins, aiding in BBB repair
Ion channels & aquaporins	AQP4	Mediates water transport across cell membranes; its dysregulation is implicated in brain edema; targeting AQP4 inhibition (e.g., with trifluoperazine) has shown promise in preclinical studies to reduce edema
	SUR1	Forms the SUR1-TRPM4 channel with TRPM4; its inhibition (e.g., with glyburide) can effectively prevent edema in preclinical models and is a potential therapeutic target
Neurotransmitters & related molecules	Glutamate	Elevated levels correlate with the severity of brain edema; exacerbates cytotoxic edema by disrupting BBB tight junctions, activating receptors leading to astrocyte swelling
	NMDARs	Overactivation during ischemia leads to massive glutamate release, calcium overload, neuronal death, and exacerbates BBB disruption, promoting brain edema
	NO	An imbalance between the protective role of endothelial nitric oxide synthase (eNOS) and the destructive role of inducible nitric oxide synthase (iNOS) can lead to BBB damage and brain edema
Other molecules	VEGF	Promotes endothelial cell proliferation and migration, but excessive levels can increase permeability and edema
	AVP	Secretion increases after stroke and may promote the development of brain edema
